# Characterization of new, efficient *Mycobacterium tuberculosis* topoisomerase-I inhibitors and their interaction with human ABC multidrug transporters

**DOI:** 10.1371/journal.pone.0202749

**Published:** 2018-09-05

**Authors:** Csilla Temesszentandrási-Ambrus, Szilárd Tóth, Rinkee Verma, Péter Bánhegyi, István Szabadkai, Ferenc Baska, Csaba Szántai-Kis, Ruben C. Hartkoorn, Mary A. Lingerfelt, Balázs Sarkadi, Gergely Szakács, László Őrfi, Valakunja Nagaraja, Sean Ekins, Ágnes Telbisz

**Affiliations:** 1 Institute of Enzymology, RCNS-HAS, Budapest, Hungary; 2 Molecular Medicine Doctoral School, Semmelweis University, Budapest, Hungary; 3 Department of Microbiology and Cell Biology, Indian Institute of Science, Bangalore, India; 4 Vichem Chemie Research Ltd., Budapest, Hungary; 5 Chemical Biology of Antibiotics, Center for Infection and Immunity, Inserm, CNRS, Institut Pasteur de Lille, Université de Lille, Lille, France; 6 Collaborations Pharmaceuticals, Inc., Raleigh, United States of America; 7 Department of Pharmaceutical Chemistry, Semmelweis University, Budapest, Hungary; Columbia University, UNITED STATES

## Abstract

Drug resistant tuberculosis (TB) is a major worldwide health problem. In addition to the bacterial mechanisms, human drug transporters limiting the cellular accumulation and the pharmacological disposition of drugs also influence the efficacy of treatment. *Mycobacterium tuberculosis* topoisomerase-I (MtTopo-I) is a promising target for antimicrobial treatment. In our previous work we have identified several hit compounds targeting the MtTopo-I by *in silico* docking. Here we expand the scope of the compounds around three scaffolds associated with potent MtTopo-I inhibition. In addition to measuring the effect of newly generated compounds on MtTopo-I activity, we characterized the compounds’ antimicrobial activity, toxicity in human cells, and interactions with human multidrug transporters. Some of the newly developed MtTopo-I inhibitors have strong antimicrobial activity and do not harm mammalian cells. Moreover, our studies revealed significant human ABC drug transporter interactions for several MtTopo-I compounds that may modify their ADME-Tox parameters and cellular effects. Promising new drug candidates may be selected based on these studies for further anti-TB drug development.

## Introduction

Multidrug resistant tuberculosis (MDR-TB) and extensively drug resistant tuberculosis (XDR-TB) are becoming a major worldwide health problem. According to the WHO database (2017), 490,000 drug resistant TB cases were identified in 2016, and an additional 110,000 cases were reported to be susceptible to isoniazid but resistant to rifampicin (RR-TB). By the end of 2016, the average proportion of MDR-TB cases, together with XDR-TB was 6.2%. In the less developed African and Asian areas the observed number of drug resistant cases is especially high. Co-morbidity of TB with other diseases, including HIV-AIDS is widely observed, and inadequate antibiotic treatment has also been shown to contribute to TB drug resistance in patients.

In addition to bacterial factors related to mutations or metabolic adaptation, antituberculotic drug resistance is also shaped by host cell factors [1–3]. Human multidrug transporters of the ATP binding cassette (ABC) superfamily protect the organism against xenobiotics and influence the passage of drugs through the cell membranes and tissue barriers. Several antituberculotic agents, including fluoroquinolones and aminoglycosides were shown to be substrates, inhibitors, or inducers of the two prominent human MDR transporters, ABCB1 and ABCG2 [1, 4–8]. These transporters were shown to play a major role in TB resistance, especially in cases when the drug treatment is prolonged [9]. MDR transporters are also present in the surface membrane of alveolar macrophages, shielding surviving *Mycobacterium tuberculosis* bacteria [10–12]. For these reasons, investigating ABC transporter interactions in the early stage of anti-TB drug development is indispensable.

To overcome bacterial resistance in tuberculosis, specific mycobacterial targets have been investigated, among them type-I DNA topoisomerases that catalyze the relaxation of negatively supercoiled DNA. As opposed to other types of microorganisms, *Mycobacterium tuberculosis* (Mtb) has a single topoisomerase-I enzyme (MtTopo-I)[13–15]. According to transposon-based knock-down experiments, MtTopo-I is essential [16], and specific inhibitors of this enzyme are considered to be antituberculotic drugs. Our previous work has shown that MtTopo-I has unique properties and a targeted inhibitor development may result in specific inhibitors [17]. The compounds investigated in our studies were synthesized by Vichem Chemie Research Ltd. and are part of a proprietary collection of designed compounds. As a proof of concept, we screened Vichem’s compound library (639 compounds) and identified 108 compounds that bind to specific sites of MtTopo-I [18, 19]. Recently, the homology model and the crystal structure of MtTopo-I has been published, opening the way for rational design of inhibitors [17, 20–24]. From among the positive hits with benzo(g)-quinoxaline, quinoxaline or styryl-benzo(g)-quinazoline scaffolds, in the present work we selected 7 compounds for further characterization. All these compounds showed significant MtTopo-I inhibitory potential. Since the interaction of antituberculotic compounds with human MDR transporters may influence general ADME-Tox properties as well as cellular drug resistance, in addition to direct MtTopo-I inhibition and bacterial growth inhibition studies we also investigated the effect of these compounds on the activity of human ABCB1 and ABCG2. We suggest that compounds exhibiting significant Mtopo-I inhibition without mammalian cell toxicity and showing an advantageous ABC transporter interaction pattern may represent new possibilities for further anti-TB drug development.

## Materials and methods

Materials were purchased from Sigma-Aldrich or Thermo-Fisher Scientific (Calcein-AM, DCV, Presto-blue reagent).

### Vichem's compound library

**Fig 1:**
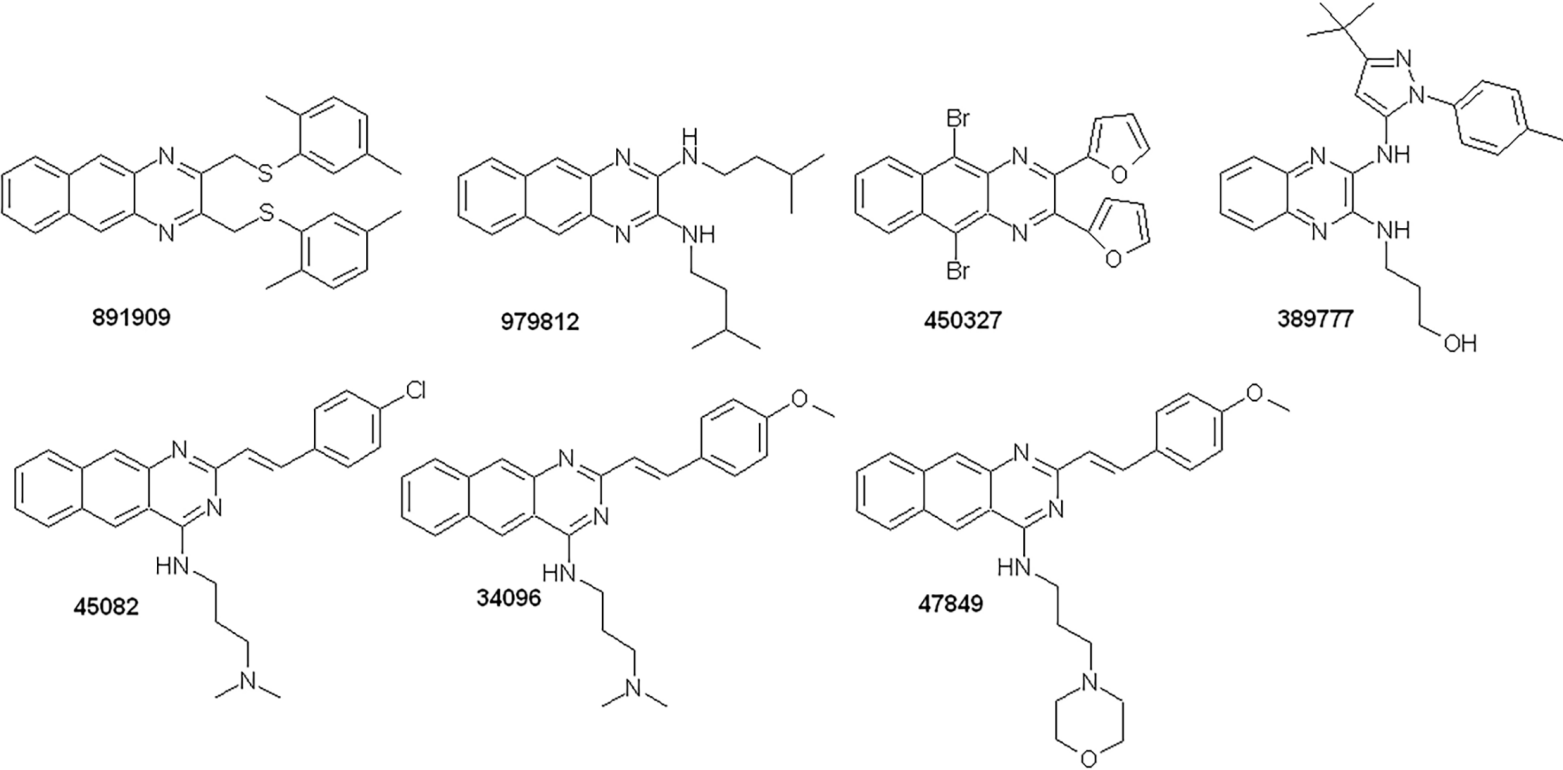
Chemical structures of the Vichem's MtTopo-I inhibitors.

The Vichem Library compounds (Fig 1) were synthesized by Vichem Chemie Research Ltd. and are part of a proprietary collection [25], (https://vichemchemie.com/nested-chemical-library-ncl/). Several Vichem Library compounds were tested previously for their MtTopo-I inhibitory potential [18], and in this paper a selected set was further characterized. The chemical structures of the selected compounds are presented in Fig 1, showing the benzo(g)-quinoxaline, quinoxaline or styryl-benzo(g)-quinazoline scaffolds. The synthesis of these compounds has been described in the Supplementary Information (S1 Fig).

### *In silico* docking studies

Docking studies were performed with the MtTopo-I structure, based on the published crystal structure (PDB ID: 5D5H), and a previous homology model, using Discovery Studio, version 4.1 (Biovia, San Diego, CA), as described previously [18]. Possible binding conformations and binding site interactions with MtTopo-I were characterized using the Schrödinger module, Induced Fit protocol, (Schrödinger Inc. Portland, OR), in which the Glide and Prime stages of searching best fit to structure had the following parameters: for the first Glide calculation, receptor and ligand Van der Waals radii were set at a default value of 50% and the maximum number of poses to report was set at 20. During the Prime stage of the calculation, residues within 5.0 Å of the ligand were refined to better accommodate the ligand. Actual docking of the ligand in the protein was performed by the Glide module in the second step of the algorithm. The top 20 poses produced by the first calculation were re-docked in the protein. Docked poses that were within 30.0 kcal/mol of the absolute lowest (relative) pose were then reconsidered, and the best two of each were kept. Box Center was chosen as centroid of residues: 48, 51, 113, 114, 115, 164, 167, 168, 171, 342, 344, 345, 346, 347, 382, 383, 384, 387, 388, 533, 534, 535, 536, and 591 [18]**. (S2 Fig)**

### DNA relaxation assay

The DNA relaxation assay of supercoiled pUC18 DNA by MtTopo-I enzyme was carried out as described previously [17, 18]. Briefly, the MtTopo-I enzyme was expressed and purified from E. coli as described in [17]. Purification of MtTopo-I enzyme was examined by SDS-PAGE. In DNA relaxation assay one unit of MtTopo-I was preincubated at 37°C for 15 min with increasing concentrations of investigated compounds from Vichem's compound library, in a reaction buffer (40 mM Tris-HCl (pH 8.0), 20 mM NaCl, 5 mM MgCl_2_, and 1 mM EDTA). In each experiment, besides the negative and solvent control, a previously established MtTopo-I inhibitor, norclomipramine, was used as positive control [21]. After preincubation, 500 ng substrate DNA was added and incubated with the enzyme at 37°C for 30 min. The samples were electrophoresed in 1.2% agarose gel for 12 h at 2.5 V/cm, stained with ethidium bromide (0.5 μg/ml), and the DNA bands were visualized using a gel documentation system (Bio-Rad, Hercules, CA, USA). The effects of compounds were tested first at 100 μM, in the next round positive hits were investigated at 50, 10, 1 and 0.1 μM concentrations. After that, further concentrations were analyzed optionally between the closest non-effective and effective concentrations, to define the concentration of the compound required for complete inhibition (S3 Fig). Assays were repeated for two to three times for each compound.

### H37Rv toxicity assay

Selected compounds from the Vichem library (Fig 1) were screened for antimicrobial effects on a replicating strain, H37Rv of *Mycobacterium tuberculosis* (Institute Pasteur, Paris), using resazurin reduction microplate assay (REMA). H37Rv was cultivated in Middlebrook 7H9 supplemented with 10% ADC, 0.2% glycerol and 0.05% Tween 80, at 37°C. For plate assays, starting culture was diluted to 0.0002 at OD600 from a log phase culture. The first screen applied 20 μM of the compounds in a 7 day-long exposure, in 384-well plate format, against replicating H37Rv, as described previously [26]. Molecules that showed more than 80% inhibition of resazurin reduction at 20 μM, were further examined to determine their minimal inhibitory concentration (MIC) by REMA, with serial dilutions of the tested compounds (20 to 0.04 μM, in two times dilution series) on 96-well plates, as described in [26] (S4 Fig). All screening was done with on plate controls (without treatment, solvent control, and rifampicin at 0.1 μg/ml, as a positive control). Plates were read on a Tecan Infinite M200. Rifampicin was always giving a Z-factor greater than 0.7. All compounds were measured at least in two independent experiments.

### ATPase activity assay for the human ABC transporters, ABCB1 and ABCG2

The human ABCB1 and ABCG2 proteins were expressed in the baculovirus-Sf9 insect cell system. Baculovirus-infected cells were harvested at 72 hours, and the cell membrane fraction was prepared by mechanical homogenization and differential centrifugation. Human ABC transporter containing membrane vesicles were used in the ATPase activity assay as described earlier [27]. Briefly, the cell membrane vesicle preparation (5 μg membrane protein/sample) was diluted in an assay buffer containing non-ABC ATPase inhibitors, and the activity assay was started by the addition of 3.3 mM MgATP. Reference substrates were used in each assay as indicated in results. The reaction was stopped by adding SDS after incubation for 20 minutes at 37°C. Vanadate-sensitive ATPase activity was calculated based on the quantification of inorganic phosphate, as determined by a colorimetric reaction detected by a VictorX3 plate reader (Perkin Elmer) at 630 nm [28]. Each compound was measured in at least three independent experiments with three parallels in each case. DMSO, used as solvent of compounds, had no effect in this assay at the applied concentration.

### Cellular transport assays for ABCB1 and ABCG2

For cellular transport assays, mammalian PLB (lymphoblastoid) cell lines, overexpressing ABCB1 or ABCG2 were used. The parental PLB cell line was purchased from ATCC, and ABCB1 or ABCG2 overexpressing cell lines were established by using lentiviral vectors for human ABC transporters as described earlier in [29]. The transport assays were carried out in 96-well plates. 1x10^5^ cells were incubated in the presence of 0.1 μM Calcein-AM or 1 μM DCV (Dye Cycler Violet) for the ABCB1 and for the ABCG2 assays, respectively [30, 31]. The assay was performed in HPMI medium (NaCl 120 mM, KCl 5 mM, MgCl_2_ 0.4 mM, CaCl_2_ 0.04 mM, HEPES 10 mM, NaHCO_3_ 10 mM, glucose 10 mM, Na_2_HPO_4_ 5 mM; pH 7.4). Fluorescence of the cells was detected using a Victor X3 plate reader (for Calcein, exc. at 485 nm, em. at 535 nm) or the Enspire 2300 (Perkin Elmer) plate reader (for DCV, exc. at 360 nm, em. at 440 nm). Kinetics of fluorescence intensity changes were analyzed to determine relative inhibition, compared to that of a reference agent, providing full inhibition of the ABC transporter. Two of the Vichem compounds examined (VCC979812 and VCC38977) were not compatible with the DCV uptake assay because of their intrinsic fluorescence, therefore for these two compounds ABCG2 transport inhibition was characterized by following mitoxantrone uptake. Mitoxantrone uptake was measured by flow cytometry (BD FACSCanto at 633 nm) by 20 minutes incubation in the presence of 7.5 μM mitoxantrone. Each compound was measured in at least three independent experiments with three parallels in each case. DMSO, used as a solvent of compounds had no effect in this assay at the applied concentration.

### Cytotoxicity assays in mammalian cell lines

The toxic effects of the selected MtTopo-I inhibitory Vichem compounds were tested in three cell lines, HFF (human foreskin fibroblast), HEK293 (human embryonic kidney) and A431 (human epidermoid carcinoma) cell lines, obtained from ATCC. Establishment of ABC transporter-expressing A431cell lines was achieved by retroviral transduction of the cells as described previously [32].

For cytotoxicity measurements the cells were seeded in a 96-well culture plate at 5,000 cells/well (A431, HEK293) or 6000 cells/well (HFF) and incubated overnight. Cells were allowed to attach to the plate for 24 hours and then serially diluted drugs were added at final concentrations ranging from 20 μM to 3 nM, except for the reference compound, irinotecan, which was tested in the range of 60 μM to 9 nM. After 72 hours of incubation viability was assessed by PrestoBlue® assay (Invitrogen). IC_50_ values were obtained by sigmoidal curve fitting in the GraphPad Prism software. All experiments were performed in triplicates and repeated at least in three independent experiments. DMSO, used as solvent of the compounds, had no effect on viability at the applied concentration.

## Results

### Compound selection

*In silico* docking studies on MtTopo-I enzyme and MtTopo-I enzyme inhibition assays were performed previously for numerous Vichem Library compounds, while in the current study we characterized a selected set of seven MtTopo-I inhibitor compounds. For the present study we have chosen the most promising *in silico* hits with benzo(g)-quinoxaline, quinoxaline, or styryl-benzo(g)-quinazoline scaffolds. In the current study, by measuring antibacterial effects, non-desirable toxic effects, and interactions with human ABC multidrug transporters, we attempted to generate a focused prescreening system for targeted drug development.

### *In silico* docking of selected compounds into the Mt Topo-I structure

As published previously, binding potentials of several compounds from the Vichem's NCL library and the FDA drug catalogue were examined by *in silico* studies that predicted binding of compounds to the MtTopo-I enzyme. These studies used a homology model of MtTopo-I, refined by a published crystal structure of MtTopo-I, and potential interaction sites were further characterized by a machine learning method [18, 24]. Several Vichem Library compounds as well as clomipramine (previously identified as an inhibitor of MtTopo-I activity), were docked in the MtTopo-I crystal structure (10.2210/pdb5d5h/pdb) to identify potential interaction sites (S2 Fig).

Representative docking results are shown in the Supplementary material. S2 Fig. shows a part of the protein structure where key interactions were observed with Arg167 and Arg114 in MtTopo-I. In the Schrodinger method, "goodness" of drug binding in the protein structure is characterized by a GlideScore that is an empirical scoring function designed to maximize separation of compounds with strong binding affinity from those with little to no binding ability. Table 1 shows GlideScores for clomipramine and for the Vichem compounds selected for more detailed biochemical characterization. All selected compounds scored reasonably well in docking studies, suggesting binding to the investigated site, and all but two of the Vichem compounds scored more favorably than clomipramine.

**Table 1:** Docking score results for selected Vichem Library compounds.

Identifier	Docking
	Glide Score (kcal/mol)
VCC340963	-5.019
VCC450822	-4.742
VCC979812	-2.901
VCC450327	-2.628
VCC389777	-2.475
VCC891909	-2.241
VCC478498	-1.614
Clomipramine	-2.383

Selected Vichem Library compounds were docked into the 5D5H crystal structure of MtTopo-I in addition to clomipramine, which has been previously identified as an inhibitor.

### Determination of MtTopo-I inhibitory potential

MtTopo-I was expressed and purified from E. coli as described previously [17]. The inhibitory effect of compounds from Vichem NCL Library on MtTopo-I enzyme activity was investigated by *in vitro* DNA relaxation assays. Norclomipramine, a previously established MtTopo-I inhibitor, was used as a positive control [21]. Concentrations required for complete inhibition of the MtTopo-I activity was determined by applying a gradually refined concentration set for a large number of compounds (Table 2, S3 Fig). Here we present data for the selected set of Vichem's compounds (Table 2). Although the MtTopo-I inhibitory potential of the selected compounds was somewhat different, all compounds completely inhibited MtTopo-I activity below 20 μM. The most potent inhibitor of this selection was VCC450327, showing complete inhibition at 0.1 μM.

**Table 2:** Effects of selected compounds from Vichem's compound library on MtTopoI activity and growth of H37Rv Mtb strain.

Identifier	Complete inhibition of MtTopoI (μM)	MIC90 of compounds in H37Rv growth test (μM)
VCC891909	7.5–10	10.4
VCC979812	10	N.I.
VCC389777	7.5–10	N.I.
VCC450327	0.1	N.I.
VCC450822	10.0	5.1
VCC340963	10–25	2.5
VCC478498	10–25	0.6

*N.I. means—no inhibition was observed up to 20 μM

Inhibition of MtTopo-I was measured by DNA relaxation assays by gradually refining the concentrations required for complete inhibition. Bands of relaxed and supercoiled DNA were visualized on agarose gels (S3 Fig). Minimum concentrations providing complete inhibition are shown in the table. The toxicity of the compounds on the virulent H37Rv Mtb strain was determined by growth inhibition tests. Growth inhibitory potential of the compounds was primarily screened at 20 μM and for the effective compounds the minimum concentration providing 90% growth inhibition (MIC90 value) was determined from dose-response viability curves (20 to 0.04 μM). The dose-response curves are presented in supplementary figures (S4 Fig).

### Antituberculotic potential of selected compounds assessed in H37Rv growth assay

Antimicrobial potential of Vichem's topoisomerase inhibitor compounds was tested in a growth inhibition assay of the virulent H37Rv mycobacterial strain, first at 20 μM concentrations. Growth of the H37Rv strain was monitored over a period of seven days. Four compounds inhibited the growth of Mtb bacteria below 20 μM, as shown in Table 2, whereas three compounds in this set, despite structural similarities, were ineffective. The MIC90 values were determined from dose-response curves, ranging from 20 to 0.04 μM concentrations (S4 Fig). The MIC90 of four examined compounds was between 0.6–10 μM (Table 2, S4 Fig), suggesting efficient *in vitro* antibacterial effect.

### Interactions of selected compounds with human ABC multidrug transporters

#### ATPase assay results

As mentioned in the introduction, ABC multidrug transporters have an important role in ADME-Tox parameters of many drugs and the detailed investigation of transporter-drug interactions is required in drug development. ABC transporter–drug interactions can be examined by various assays that show such interactions from different aspects. ATPase activity and drug transport are coupled processes in human ABC transporters, and most interacting drugs influence the ATPase activity in a concentration dependent manner. Measuring the inhibition of the active transport of a fluorescent substrate by a test compound is a more sensitive assay, while does not directly show if the test compound is a competing transported substrate or a non-transported inhibitor. Finally, cellular toxicity can also be modified by the expression of ABC drug transporters, and for assessing transporter-drug interactions viability assays can be performed in transporter expressing cells.

In this study ATPase measurements were performed in isolated Sf9 membrane vesicles prepared from human ABC multidrug transporters expressing cells, containing high levels of the human transporters. In the Sf9 membrane ATPase assay, measuring the vanadate-sensitive ATPase activity of the transporter provides a simple but efficient tool to characterize the effects of various drugs [28, 33]. This ATPase activity often has a complex dose-response curve in the presence of drug substrates, and a considerable basal ATPase activity (without any given drug) also complicates the assessment of drug effects.

**Fig 2:**
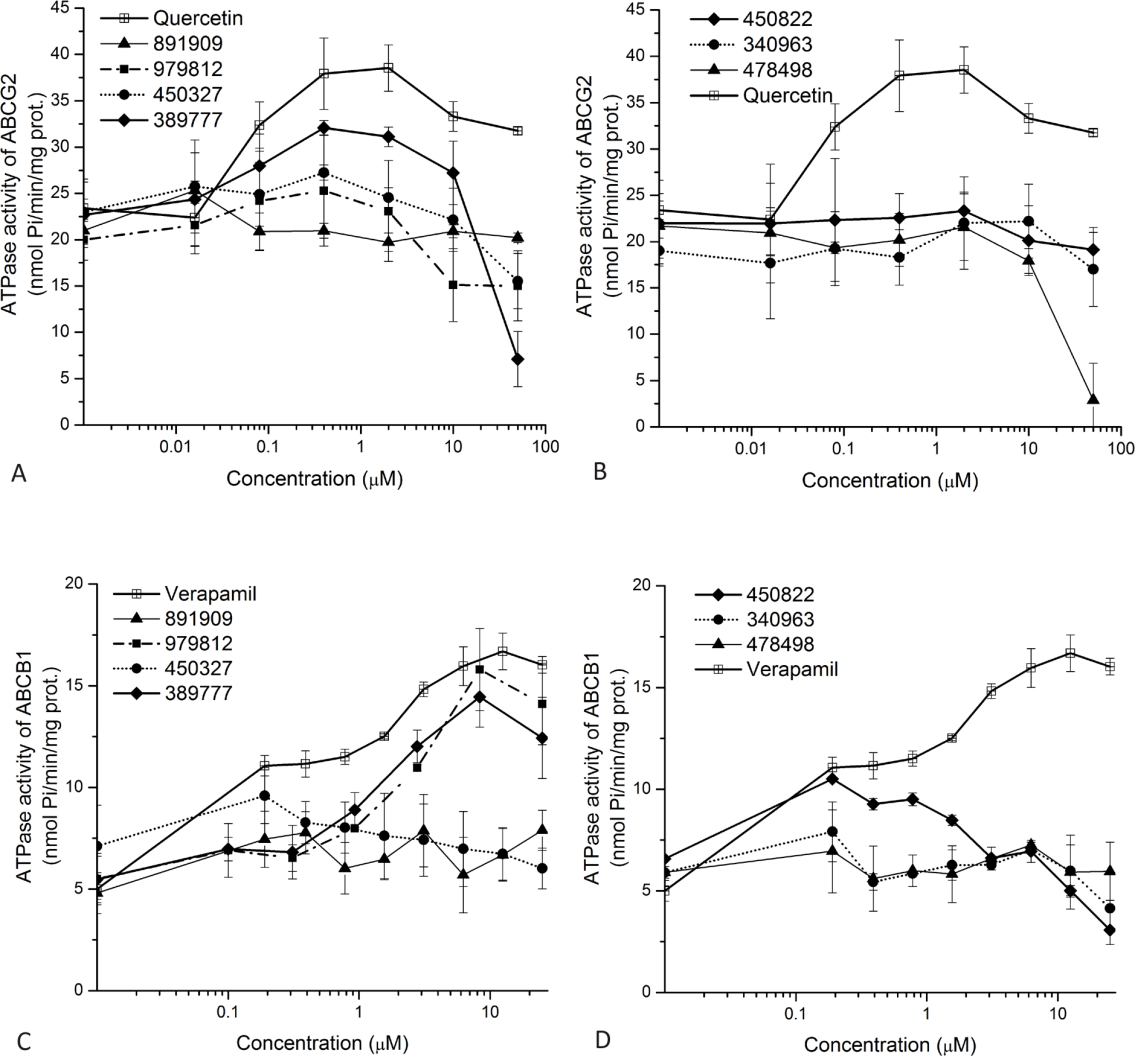
Effects of the Vichem MtTopo-I inhibitors on the ATPase activity of the human ABCG2 and ABCB1 transporters. Purified Sf9 membrane vesicles containing ABCG2 (Upper panels, A and B) or ABCB1 proteins (lower panels, C and D) were used in ATPase assays as described in the Methods section. Vanadate sensitive (ABC transporter related) ATPase activities were measured in the presence of investigated MtTopo-I inhibitors. Compound identity numbers are shown in the Figure. For the sake of visibility, compounds were divided into two groups. At zero compound levels, basal ATPase activity of the ABC transporter, measured without the addition of any drug, is shown. Quercetin (for ABCG2) and verapamil (for ABCB1) were used as reference substrates that are known transported substrates and activate the ATPase activity of the given transporter.

As shown in Fig 2, we examined the effects of the selected Vichem compounds by following dose-response curves of ATPase activity. The V_max_ values, less informative for these interactions, were determined from curves presented in Supplementary file (S5 Fig). We found that VCC389777 significantly stimulated the ABCG2-ATPase activity, while three compounds, VCC389777, VCC979812, and VCC450822 had a well measurable stimulatory effect on the ABCB1-ATPase activity, suggesting the active transport of these compounds. Compound VCC450822 showed a slight stimulation of the ABCB1-ATPase activity at lower concentrations, while strongly inhibited ATPase activity at higher concentrations. This behavior is typical for slowly transported substrates and/or inhibitors of the transporters [33].

#### Transport assay results

**Fig 3:**
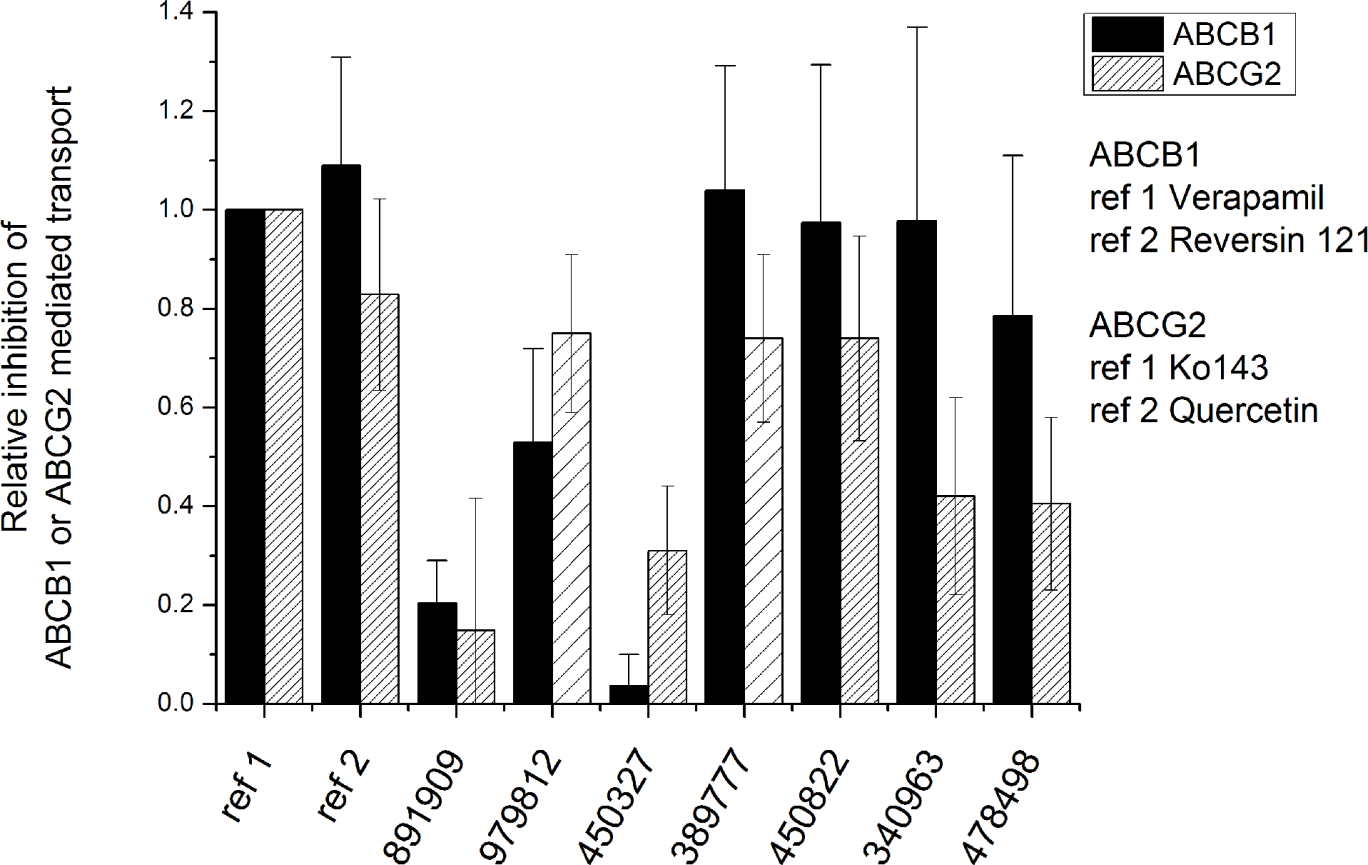
Effects of the Vichem MtTopoI inhibitors on the transport activity of human ABCG2 and ABCB1 transporters. Effects of the compounds on the transport activity of human ABCG2 and ABCB1 proteins were measured in transporter-overexpressing human PLB cells. Accumulation of transporter-specific probe substrates (Calcein-AM for ABCB1 and DCV for ABCG2) was measured in the presence of MtTopo-I inhibitor compounds (5 μM). The relative inhibitory effects of MtTopo-I inhibitors were determined by using a reference substrate, providing maximum inhibition (20 μM verapamil or 5 μM reversin 121 for ABCB1; and 5 μM Ko143 or 5μM quercetin for ABCG2). Compounds VCC979812 and VCC38977 were not compatible with the DCV uptake assay. In these cases (columns with sparse pattern on the figure) ABCG2 transport inhibition was characterized by inhibition of mitoxantrone extrusion, measured after 20 minutes of incubation in the same cell types with the same reference inhibitors. Relative inhibition values higher than 0.2 are statistically significant, based on comparison to control (0) level, by the Student's t-test (p<0.05).

The selected Vichem compounds were further characterized in whole cell transport assays. In these experiments we used transporter-overexpressing human PLB cells and measured the uptake of various fluorescent substrates of ABCG2 and ABCB1. This type of transport assays is commonly used to explore potential transporter-drug interactions. We found that, except for VCC891909, all the compounds analyzed showed significant interactions with ABCG2 and/or ABCB1 (Fig 3). VCC450327 was found to only moderately influence the ABCG2 mediated transport, while having no effect on ABCB1 mediated transport.

### Toxicity of the selected Vichem compounds in human cells

Toxicity was evaluated by using several human cell lines. HFF (human foreskin fibroblast) and HEK293 cells are non-tumor cell lines, whereas A431 cells are derived from a carcinoma. To examine the potential influence of ABC transporters on the human cell toxicity of the investigated compounds, we have also applied ABCB1- and ABCG2-overexpressing A431 cell lines. Irinotecan, a known human topoisomerase inhibitor was used as a reference compound, as this drug is a transported substrate of both ABCG2 and ABCB1 [34].

Table 3 shows that VCC891909 has no toxicity in any of the investigated cell lines, but the other investigated compounds showed variable toxicity. VCC480522, VCC340963 and VCC478498 were toxic (with IC50 values below 5 μM) in the parental A431 and HFF cells, but no difference was observed in the toxicity or the IC_50_ values between the parental and ABCB1 or ABCG2 expressing A431 cells (Table 3).

**Table 3:** Cytotoxicity of the selected MtTopoI inhibitor compounds in human cell lines.

Identifier	Toxicity in HFF cell line cell line, IC_50_ (μM)	Toxicity in HEK293 cell line cell line, IC_50_ (μM)	Toxicity in parental A431 cell line, IC_50_ (μM)	Resistance modulation by ABCB1 (fold increase in IC_50_)	Resistance modulation by ABCG2 (fold increase in IC_50_)
VCC891909	Non-toxic+	Non-toxic	Non-toxic	NA*	NA
VCC979812	Toxic, 20	Non-toxic	Toxic, 19±2	1.1±0.08	1±0.1
VCC389777	Non-toxic	Non-toxic	Non-toxic	NA	NA
VCC450327	Non-toxic	Non-toxic	Non-toxic	NA	NA
VCC450822	Toxic, 2.5–5	Toxic, 10–20	Toxic, 3.5±0.2	0.97±0.05	1.0±0.03
VCC340963	Toxic, 2.5–5	Toxic, 10–20	Toxic, 3.23±0.4	0.77±0.3	0.9±0.32
VCC478498	Toxic, 2.5–5	Toxic, 10–20	Toxic, 3.03±0.61	0.88±0.06	0.79±0.14
Irinotecan	Non-toxic	Toxic, 2.5	Toxic, 8.6±0.5	2.60±0.01	4.12±0.16

* NA: Not applicable

Cytotoxicity of the compounds was measured in a 72-hours assay in the presence of the indicated compounds. Viability was assessed using the Presto blue® assay. Irinotecan was used as a reference compound. IC_50_ values presented in the table are determined from dose-response viability curves (not shown). Compounds were tested from 20 μM down to 3 nM. Non-toxic phrase in the table means that the compound is not toxic up to 20 μM.

## Discussion

The increasing number of drug resistant TB cases provides a strong incentive to find new, effective antituberculotic agents, potentially avoiding bacterial drug-resistance. Targeting MtTopo-I, an essential and unique mycobacterial DNA repair enzyme offers a promising possibility in this regard [22]. The docking of candidate inhibitor molecules in the recently described crystal structure of MtTopo-I [24] may help to design new antibacterial agents. Since imipramine and clomipramine were shown to be MtTopo-I inhibitors, these molecules served as model drugs for *in silico* docking studies [18]. A compound library of Vichem was screened by such docking studies, and a selected set of compounds was characterized in biological assays [18]. In this paper we show a detailed biological characterization of seven promising topoisomerase inhibitors, selected from Vichem compounds, designed with three different scaffolds.

Optimization of the screening of designed antimycobacterial topoisomerase inhibitor compounds, in addition to the investigation of the antimycobacterial effect, involves steps to find molecules that have limited toxicity in mammalian cells, and a known pattern of human ABC multidrug transporter interactions. Human ABC multidrug efflux transporters are expressed in major tissue barriers and in numerous cell types, including macrophages, thus these transporters significantly modulate ADME-toxicity profiles and cellular effectiveness for a wide variety of drugs [33, 35]. The human ABCG2 and ABCB1 proteins are the key multidrug transporters which have been documented to significantly reduce the cellular uptake and modulate the ADME-Tox parameters of various antibiotics and topoisomerase inhibitors [35]. If a drug candidate is an efficiently transported substrate of these proteins, this generally reduces the oral bioavailability (i.e. intestinal absorption) and the cellular entry of the molecule. In contrast, an inhibitory effect on these drug transporters may facilitate both the absorption and cellular availability of the given compound, as well as that of other, co-administered antimicrobial agents.

In order to explore the direct human cellular toxicity of the selected Vichem compounds, here we used two non-tumor cell lines (HFF and HEK293) and the A431 tumor cell line. A431 is a rapidly growing cell type with known sensitivity to topoisomerase inhibitors. In addition, we also examined drug-resistant derivatives of this cell line to show the potential role of ABCB1 and ABCG2 transporter in toxicity. We found that, although the antimycobacterial effects were promising, some of the Vichem compounds examined here were toxic in at least one of the examined cell line, with IC_50_ values below 5 μM. Therefore, these compounds probably cannot be considered as promising candidates for *in vivo* human applications.

For further selection of drug candidates, we examined the selected Vichem compounds for ABC transporter interaction in multiple *in vitro* assays (summarized in S6 Fig), aiming for the selection of lead compounds with favorable transporter interaction pattern. In the ATPase activity assay for ABCG2 and ABCB1 transporters, in concentrations of 0.1–10 μM, several compounds (VCC389777 for ABCG2, and VCC38977, VCC450822, VCC979812 for ABCB1) slightly increased the ATPase activity of either the ABCG2 or ABCB1 protein, indicating that these compounds are likely recognized as transported substrates. In contrast, for ABCG2 in some cases (VCC478498) we obtained only an inhibition of the ATPase activity at high (50 μM) concentrations, and some of the compounds had no measurable effect on the transporter ATPase activity (VCC891909, VCC340963).

In the direct ABC transporter inhibition assays, providing increased sensitivity but without information about the type of interaction, we found that most of the examined compounds strongly inhibited both ABCG2 and ABCB1 mediated transport of a model compound at concentrations around 5 μM. VCC891909 had no such transporter inhibitory effect, while VCC450327 had only a slight effect at 5 μM, and only on the ABCG2 transporter. It is worth mentioning that when we used the drug-resistant, ABCB1 or ABCG2 expressing A431 cell lines, we did not find any significant modulation of the toxicity profiles by these ABC transporters. This finding suggests that the Vichem compounds are slowly transported compounds or rather ABC transporter inhibitors—it should be mentioned that none of these assays clearly distinguish transported substrates and inhibitors, as slowly transported substrates have inhibitory effects at higher concentrations.

Taken together, our data show that VCC979812, VCC450822, VCC340936 and VCC478498 showed interactions with the human ABC transporters, while these compounds were not efficiently extruded by these transporters. VCC891909 had no toxicity in any of the investigated cell lines (HFF, HEK293 and A431). Three other compounds (VCC891909, VCC450327 and VCC389777) were non-toxic in human cells, while may act as direct transporter inhibitors (see S5 Fig).

As a summary, although VCC450822, VCC340963 and VCC478498 effectively inhibited the growth of H37Rv mycobacteria, these compounds were toxic in human cell lines, thus may not be candidates for further human drug development. This toxicity was not significantly affected by the expression of the ABC multidrug transporters. In contrast, VCC891909 showed efficient topoisomerase inhibition, Mtb growth inhibitory effect, and no mammalian cell toxicity without a significant human ABC transporter interaction. Therefore, this compound may be an especially favorable candidate for further antimycobacterial drug development.

## Supporting information

S1 Fig Chemical synthesis scheme of the selected Vichem compounds. The synthetic route of the VCC891909, VCC979812 and VCC450327 is described in WO2002094796 patent (as examples 63, 194 and 369).
(PDF)

S2 Fig Docking of topoisomerase inhibitors in the crystal structure 5D5H (PDB ID) of Mt Topo-I. 2D structures highlight the key interactions.
(PDF)

S3 Fig Representative pictures for DNA relaxation assay results presented in Table 2. (PDF)

S4 Fig Toxicity of the investigated compounds in H37Rv Mtb strains. (PDF)

S5 Fig Effects of the Vichem MtTopo-I inhibitors on the ATPase activity of the human ABCG2 and ABCB1 transporters. (PDF)

S6 Fig Summary table for the results of the assays. (PDF)

## Abbreviations

ADME-Tox: Absorption-Distribution-Metabolism-Excretion-Toxicity
ABC transporter: ATP Binding Cassette Transporter
DCV: Dye cycler violet
Mtb: Mycobacterium tuberculosis
MDR: multidrug resistance
MtTopo-I: topoisomerase I of *Mycobacterium tuberculosis*
TB: Tuberculosis

## References

[1] Fonseca JD, Knight GM, McHugh TD. The complex evolution of antibiotic resistance in Mycobacterium tuberculosis. International journal of infectious diseases: IJID: official publication of the International Society for Infectious Diseases. 2015;32:94–100. Epub 2015/03/27. 10.1016/j.ijid.2015.01.014 . 25809763

[2] Song L, Wu X. Development of efflux pump inhibitors in antituberculosis therapy. International journal of antimicrobial agents. 2016;47(6):421–9. Epub 2016/05/24. 10.1016/j.ijantimicag.2016.04.007 . 27211826

[3] Te Brake LH, Russel FG, van den Heuvel JJ, de Knegt GJ, de Steenwinkel JE, Burger DM, et al Inhibitory potential of tuberculosis drugs on ATP-binding cassette drug transporters. Tuberculosis (Edinburgh, Scotland). 2016;96:150–7. Epub 2015/12/20. 10.1016/j.tube.2015.08.004 . 26682943

[4] Ando T, Kusuhara H, Merino G, Alvarez AI, Schinkel AH, Sugiyama Y. Involvement of breast cancer resistance protein (ABCG2) in the biliary excretion mechanism of fluoroquinolones. Drug metabolism and disposition: the biological fate of chemicals. 2007;35(10):1873–9. Epub 2007/07/20. 10.1124/dmd.107.014969 . 17639028

[5] Brillault J, De Castro WV, Couet W. Relative contributions of active mediated transport and passive diffusion of fluoroquinolones with various lipophilicities in a Calu-3 lung epithelial cell model. Antimicrobial agents and chemotherapy. 2010;54(1):543–5. Epub 2009/10/14. 10.1128/AAC.00733-09 ; PubMed Central PMCID: PMCPmc2798514. 19822706 PMC2798514

[6] Haslam IS, Wright JA, O'Reilly DA, Sherlock DJ, Coleman T, Simmons NL. Intestinal ciprofloxacin efflux: the role of breast cancer resistance protein (ABCG2). Drug metabolism and disposition: the biological fate of chemicals. 2011;39(12):2321–8. Epub 2011/09/21. 10.1124/dmd.111.038323 ; PubMed Central PMCID: PMCPmc3226371. 21930826 PMC3226371

[7] Merino G, Alvarez AI, Pulido MM, Molina AJ, Schinkel AH, Prieto JG. Breast cancer resistance protein (BCRP/ABCG2) transports fluoroquinolone antibiotics and affects their oral availability, pharmacokinetics, and milk secretion. Drug metabolism and disposition: the biological fate of chemicals. 2006;34(4):690–5. Epub 2006/01/26. 10.1124/dmd.105.008219 . 16434544

[8] Zumla A, Raviglione M, Hafner R, von Reyn CF. Tuberculosis. The New England journal of medicine. 2013;368(8):745–55. Epub 2013/02/22. 10.1056/NEJMra1200894 . 23425167

[9] Hartkoorn RC, Chandler B, Owen A, Ward SA, Bertel Squire S, Back DJ, et al Differential drug susceptibility of intracellular and extracellular tuberculosis, and the impact of P-glycoprotein. Tuberculosis (Edinburgh, Scotland). 2007;87(3):248–55. Epub 2007/01/30. 10.1016/j.tube.2006.12.001 . 17258938

[10] Scheffer GL, Pijnenborg AC, Smit EF, Muller M, Postma DS, Timens W, et al Multidrug resistance related molecules in human and murine lung. Journal of clinical pathology. 2002;55(5):332–9. Epub 2002/05/03. ; PubMed Central PMCID: PMCPmc1769658. 11986335 10.1136/jcp.55.5.332 PMC1769658

[11] van der Deen M, de Vries EG, Timens W, Scheper RJ, Timmer-Bosscha H, Postma DS. ATP-binding cassette (ABC) transporters in normal and pathological lung. Respiratory research. 2005;6:59 Epub 2005/06/22. 10.1186/1465-9921-6-59 ; PubMed Central PMCID: PMCPmc1200430. 15967026 PMC1200430

[12] van der Valk P, van Kalken CK, Ketelaars H, Broxterman HJ, Scheffer G, Kuiper CM, et al Distribution of multi-drug resistance-associated P-glycoprotein in normal and neoplastic human tissues. Analysis with 3 monoclonal antibodies recognizing different epitopes of the P-glycoprotein molecule. Annals of oncology: official journal of the European Society for Medical Oncology. 1990;1(1):56–64. Epub 1990/01/01. 1706611

[13] Ahmed W, Menon S, Godbole AA, Karthik PV, Nagaraja V. Conditional silencing of topoisomerase I gene of Mycobacterium tuberculosis validates its essentiality for cell survival. FEMS microbiology letters. 2014;353(2):116–23. Epub 2014/03/07. 10.1111/1574-6968.12412 . 24593153

[14] Cole ST, Brosch R, Parkhill J, Garnier T, Churcher C, Harris D, et al Deciphering the biology of Mycobacterium tuberculosis from the complete genome sequence. Nature. 1998;393(6685):537–44. Epub 1998/06/20. 10.1038/31159 . 9634230

[15] Ravishankar S, Ambady A, Awasthy D, Mudugal NV, Menasinakai S, Jatheendranath S, et al Genetic and chemical validation identifies Mycobacterium tuberculosis topoisomerase I as an attractive anti-tubercular target. Tuberculosis (Edinburgh, Scotland). 2015;95(5):589–98. Epub 2015/06/16. 10.1016/j.tube.2015.05.004 . 26073894

[16] Guilhot C, Otal I, Van Rompaey I, Martin C, Gicquel B. Efficient transposition in mycobacteria: construction of Mycobacterium smegmatis insertional mutant libraries. Journal of bacteriology. 1994;176(2):535–9. Epub 1994/01/01. ; PubMed Central PMCID: PMCPmc205082. 8288551 10.1128/jb.176.2.535-539.1994 PMC205082

[17] Godbole AA, Leelaram MN, Bhat AG, Jain P, Nagaraja V. Characterization of DNA topoisomerase I from Mycobacterium tuberculosis: DNA cleavage and religation properties and inhibition of its activity. Archives of biochemistry and biophysics. 2012;528(2):197–203. Epub 2012/10/23. 10.1016/j.abb.2012.10.004 . 23085346

[18] Ekins S, Godbole AA, Keri G, Orfi L, Pato J, Bhat RS, et al Machine learning and docking models for Mycobacterium tuberculosis topoisomerase I. Tuberculosis (Edinburgh, Scotland). 2017;103:52–60. Epub 2017/02/27. 10.1016/j.tube.2017.01.005 . 28237034

[19] Sipos A, Pató J, Székely R, Hartkoorn RC, Kékesi L, Őrfi L, et al Lead selection and characterization of antitubercular compounds using the Nested Chemical Library. Tuberculosis. 2015;95:S200–S6. 10.1016/j.tube.2015.02.028 25801335

[20] Chaudhari K, Surana S, Jain P, Patel HM. Mycobacterium Tuberculosis (MTB) GyrB inhibitors: An attractive approach for developing novel drugs against TB. European journal of medicinal chemistry. 2016;124:160–85. Epub 2016/08/30. 10.1016/j.ejmech.2016.08.034 . 27569197

[21] Godbole AA, Ahmed W, Bhat RS, Bradley EK, Ekins S, Nagaraja V. Targeting Mycobacterium tuberculosis topoisomerase I by small-molecule inhibitors. Antimicrobial agents and chemotherapy. 2015;59(3):1549–57. Epub 2014/12/24. 10.1128/AAC.04516-14 ; PubMed Central PMCID: PMC4325804. 25534741 PMC4325804

[22] Nagaraja V, Godbole AA, Henderson SR, Maxwell A. DNA topoisomerase I and DNA gyrase as targets for TB therapy. Drug discovery today. 2017;22(3):510–8. Epub 2016/11/20. 10.1016/j.drudis.2016.11.006 . 27856347

[23] Sandhaus S, Annamalai T, Welmaker G, Houghten RA, Paz C, Garcia PK, et al Small-Molecule Inhibitors Targeting Topoisomerase I as Novel Antituberculosis Agents. Antimicrobial agents and chemotherapy. 2016;60(7):4028–36. Epub 2016/04/27. 10.1128/AAC.00288-16 ; PubMed Central PMCID: PMCPmc4914652. 27114277 PMC4914652

[24] Tan K, Cao N, Cheng B, Joachimiak A, Tse-Dinh YC. Insights from the Structure of Mycobacterium tuberculosis Topoisomerase I with a Novel Protein Fold. Journal of molecular biology. 2016;428(1):182–93. Epub 2015/12/15. 10.1016/j.jmb.2015.11.024 ; PubMed Central PMCID: PMCPmc4738035. 26655023 PMC4738035

[25] Keri G, Szekelyhidi Z, Banhegyi P, Varga Z, Hegymegi-Barakonyi B, Szantai-Kis C, et al Drug discovery in the kinase inhibitory field using the Nested Chemical Library technology. Assay and drug development technologies. 2005;3(5):543–51. Epub 2005/11/25. 10.1089/adt.2005.3.543 . 16305311

[26] Sala C, Dhar N, Hartkoorn RC, Zhang M, Ha YH, Schneider P, et al Simple model for testing drugs against nonreplicating Mycobacterium tuberculosis. Antimicrobial agents and chemotherapy. 2010;54(10):4150–8. Epub 2010/08/04. 10.1128/AAC.00821-10 ; PubMed Central PMCID: PMC2944619. 20679505 PMC2944619

[27] Telbisz A, Muller M, Ozvegy-Laczka C, Homolya L, Szente L, Varadi A, et al Membrane cholesterol selectively modulates the activity of the human ABCG2 multidrug transporter. Biochimica Et Biophysica Acta-Biomembranes. 2007;1768(11):2698–713. ISI:000251493700006. 10.1016/j.bbamem.2007.06.026 17662239

[28] Sarkadi B, Price EM, Boucher RC, Germann UA, Scarborough GA. Expression of the human multidrug resistance cDNA in insect cells generates a high activity drug-stimulated membrane ATPase. The Journal of biological chemistry. 1992;267(7):4854–8. . 1347044

[29] Ujhelly O, Ozvegy C, Varady G, Cervenak J, Homolya L, Grez M, et al Application of a human multidrug transporter (ABCG2) variant as selectable marker in gene transfer to progenitor cells. Human gene therapy. 2003;14(4):403–12. Epub 2003/03/28. 10.1089/104303403321209005 . 12659681

[30] Nerada Z, Hegyi Z, Szepesi A, Toth S, Hegedus C, Varady G, et al Application of fluorescent dye substrates for functional characterization of ABC multidrug transporters at a single cell level. Cytometry Part A: the journal of the International Society for Analytical Cytology. 2016 10.1002/cyto.a.22931 . 27602881

[31] Homolya L, Hollo M, Muller M, Mechetner EB, Sarkadi B. A new method for a quantitative assessment of P-glycoprotein-related multidrug resistance in tumour cells. Br J Cancer. 1996;73(7):849–55. . 8611394 10.1038/bjc.1996.151 PMC2074264

[32] Elkind NB, Szentpetery Z, Apati A, Ozvegy-Laczka C, Varady G, Ujhelly O, et al Multidrug transporter ABCG2 prevents tumor cell death induced by the epidermal growth factor receptor inhibitor Iressa (ZD1839, Gefitinib). Cancer Res. 2005;65(5):1770–7. 10.1158/0008-5472.CAN-04-3303 . 15753373

[33] Szakacs G, Varadi A, Ozvegy-Laczka C, Sarkadi B. The role of ABC transporters in drug absorption, distribution, metabolism, excretion and toxicity (ADME-Tox). Drug discovery today. 2008;13(9–10):379–93. Epub 2008/05/13. 10.1016/j.drudis.2007.12.010 . 18468555

[34] Smith NF, Figg WD, Sparreboom A. Pharmacogenetics of irinotecan metabolism and transport: an update. Toxicology in vitro: an international journal published in association with BIBRA. 2006;20(2):163–75. Epub 2005/11/08. 10.1016/j.tiv.2005.06.045 . 16271446

[35] Sarkadi B, Homolya L, Szakacs G, Varadi A. Human multidrug resistance ABCB and ABCG transporters: participation in a chemoimmunity defense system. Physiol Rev. 2006;86(4):1179–236. 10.1152/physrev.00037.2005 . 17015488

