# Supplementary material for: Characterization of new, efficient Mycobacterium tuberculosis topoisomerase-I inhibitors and their interaction with human ABC multidrug transporters
Source: PLoS One. 2018 Sep 5;13(9):e0202749. doi: 10.1371/journal.pone.0202749 (PMC6124754; doi:10.1371/journal.pone.0202749)
Supplement: S1 Fig — The synthetic route of the VCC891909, VCC979812 and VCC450327 is described in WO2002094796 patent (as examples 63, 194 and 369). (PDF) [file pone.0202749.s001.pdf]

S1 Fig. Chemical synthesis scheme of selected Vichem compounds

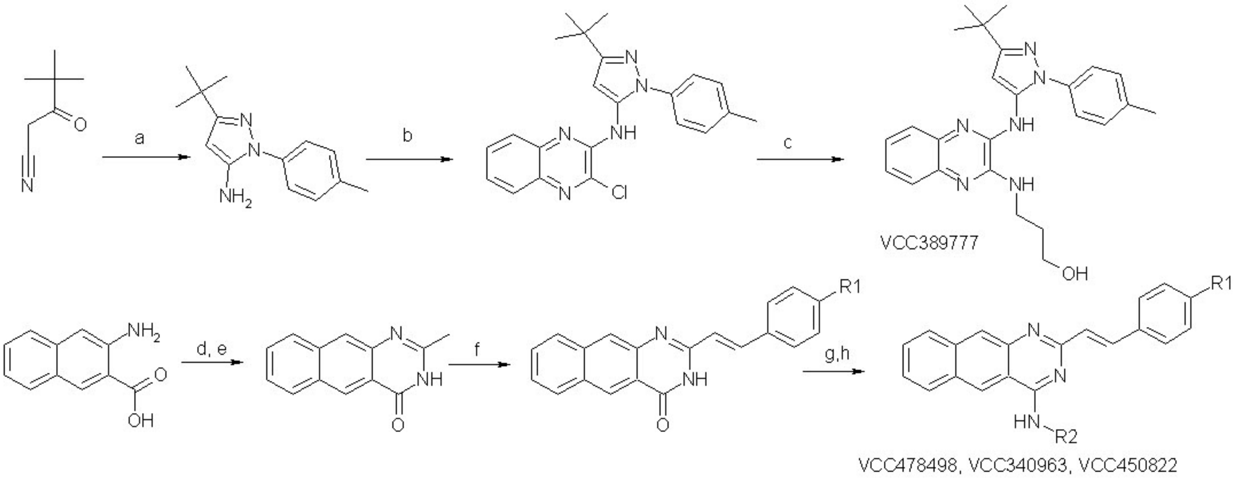

Reagents and conditions. a. p-Tolyl-hydrazine, IPA, reflux; b. 2,3-Dichloro-quinoxaline, NaH, DMSO, rt; c. 3-Amino-propan-1-ol, IPA, reflux; d. Acetic anhydride, reflux; e. Concentrated ammonium hydroxide, rt; f. 4-substituted benzaldehyde, microwave, 190ºC; g. Phosphorous oxychloride, 90ºC; h. Appropiate amine, dioxan, DIPEA, 80 ºC.

The synthetic route of the VCC891909, VCC979812 and VCC450327 is described in WO2002094796 patent (as examples 63, 194 and 369).
