# Supplementary material for: Characterization of new, efficient Mycobacterium tuberculosis topoisomerase-I inhibitors and their interaction with human ABC multidrug transporters
Source: PLoS One. 2018 Sep 5;13(9):e0202749. doi: 10.1371/journal.pone.0202749 (PMC6124754; doi:10.1371/journal.pone.0202749)
Supplement: S2 Fig — 2D structures highlight the key interactions. (PDF) [file pone.0202749.s002.pdf]

S2 Fig. Docking of topoisomerase inhibitors in the crystal structure 5D5H (PDB ID) of Mt Topo-I. 2D structures highlight the key interactions.

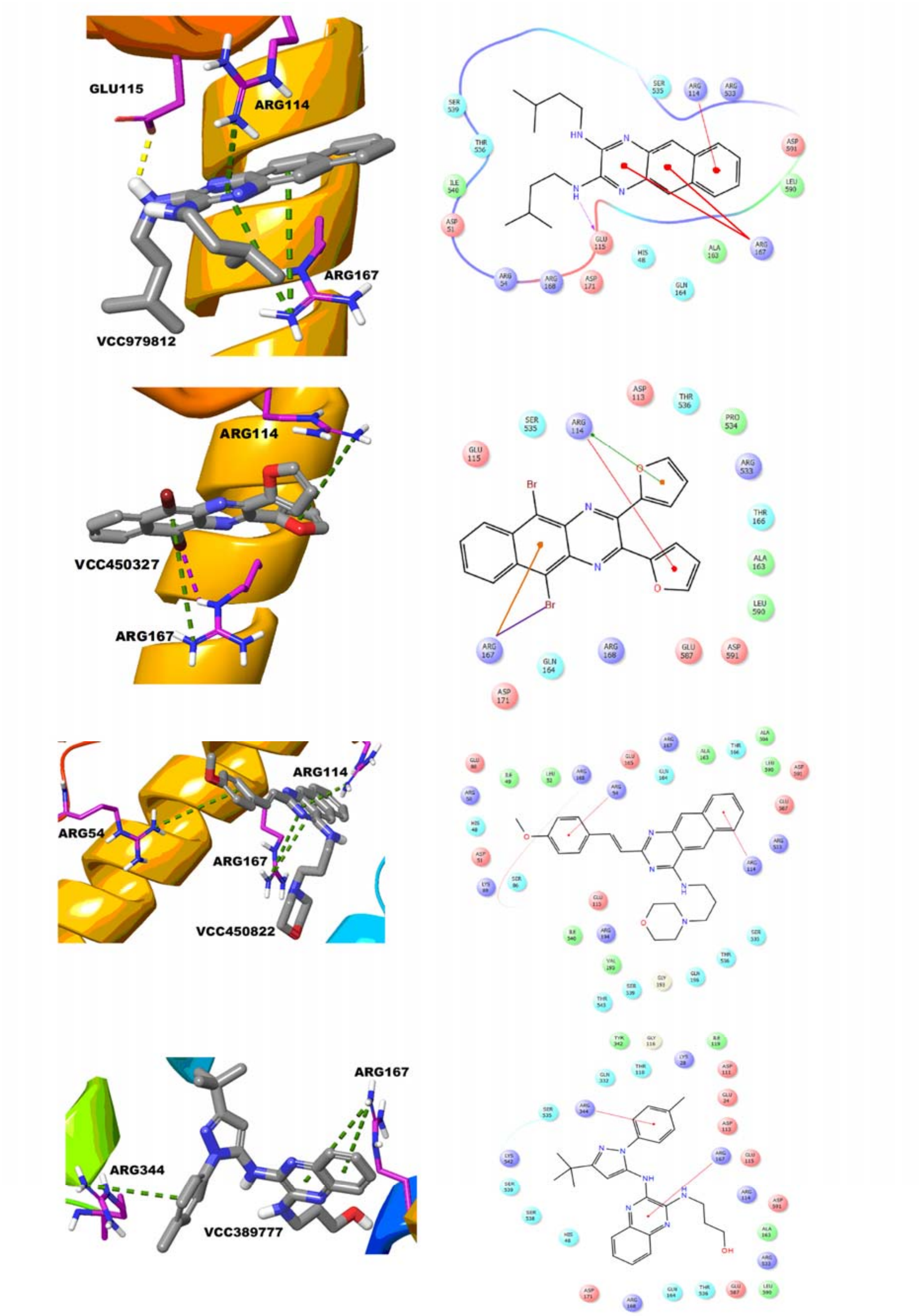

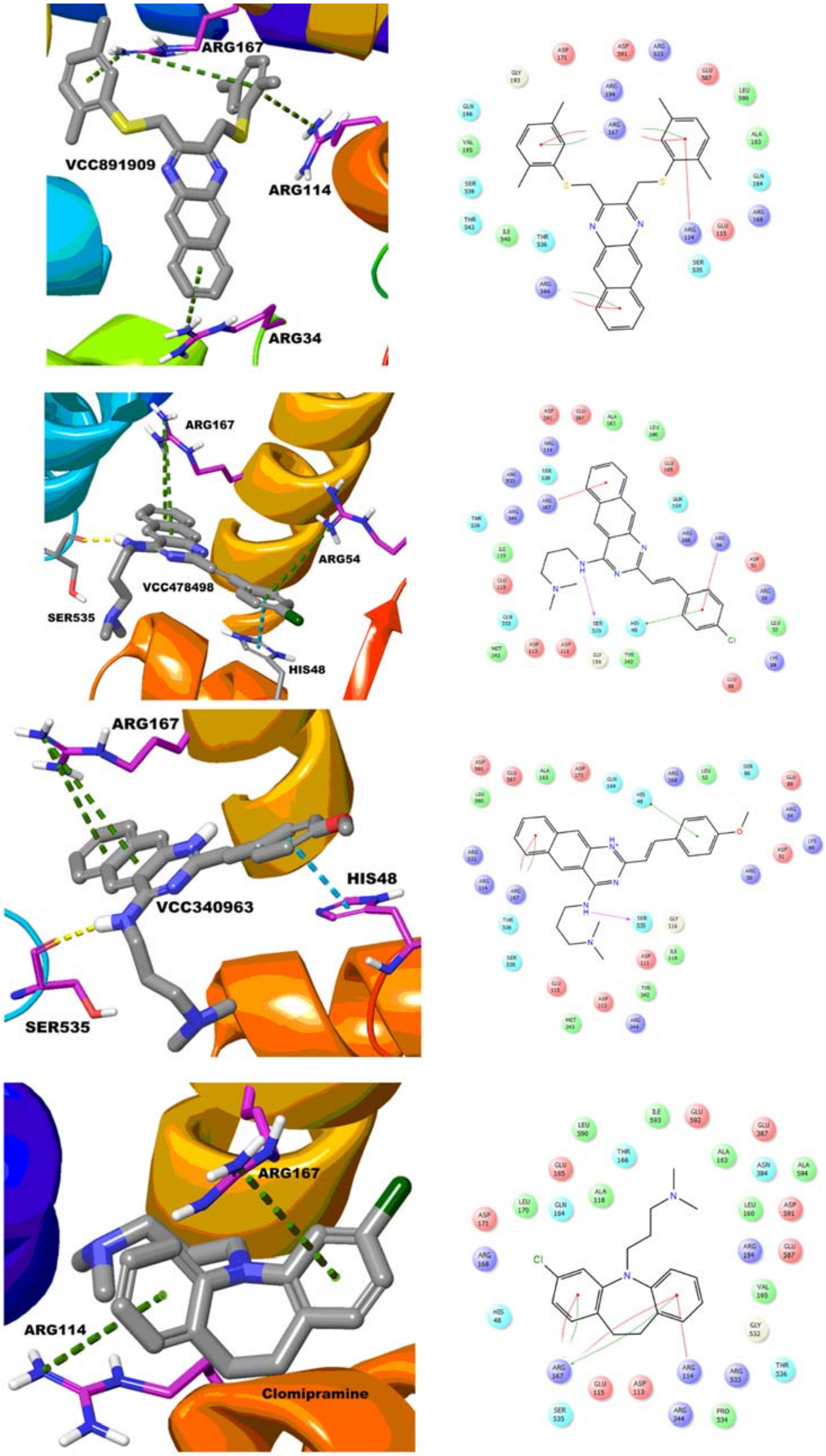
ARG167
VCC891909
ARG114
ARG34
ARG167
ARG54
VCC478498
SER535
HIS48
ARG167
VCC340963
HIS48
SER535
ARG167
ARG114
Clomipramine
