# Supplementary material for: Characterization of new, efficient Mycobacterium tuberculosis topoisomerase-I inhibitors and their interaction with human ABC multidrug transporters
Source: PLoS One. 2018 Sep 5;13(9):e0202749. doi: 10.1371/journal.pone.0202749 (PMC6124754; doi:10.1371/journal.pone.0202749)

S3 Fig. Representative pictures for DNA relaxation assay results presented in Table 2. Inhibition of MtTopo-I by the investigated compounds was measured by DNA relaxation assays by gradually refining the concentrations required for complete inhibition of purified MtTopo-I. Bands of relaxed and supercoiled DNA were visualized on agarose gels as described in the Methods section. Norclomipramine (N; 0.1 uM) was used as a positive control for inhibition. DMSO (2.5%) was used in all the reactions as solvent control (S). C; supercoiled pUC18 control, (0) non-inhibited MtTopo-I control. In the representative pictures compounds examined in this study are indicated by code numbers. Asterisks label positive hits in the screen sets.

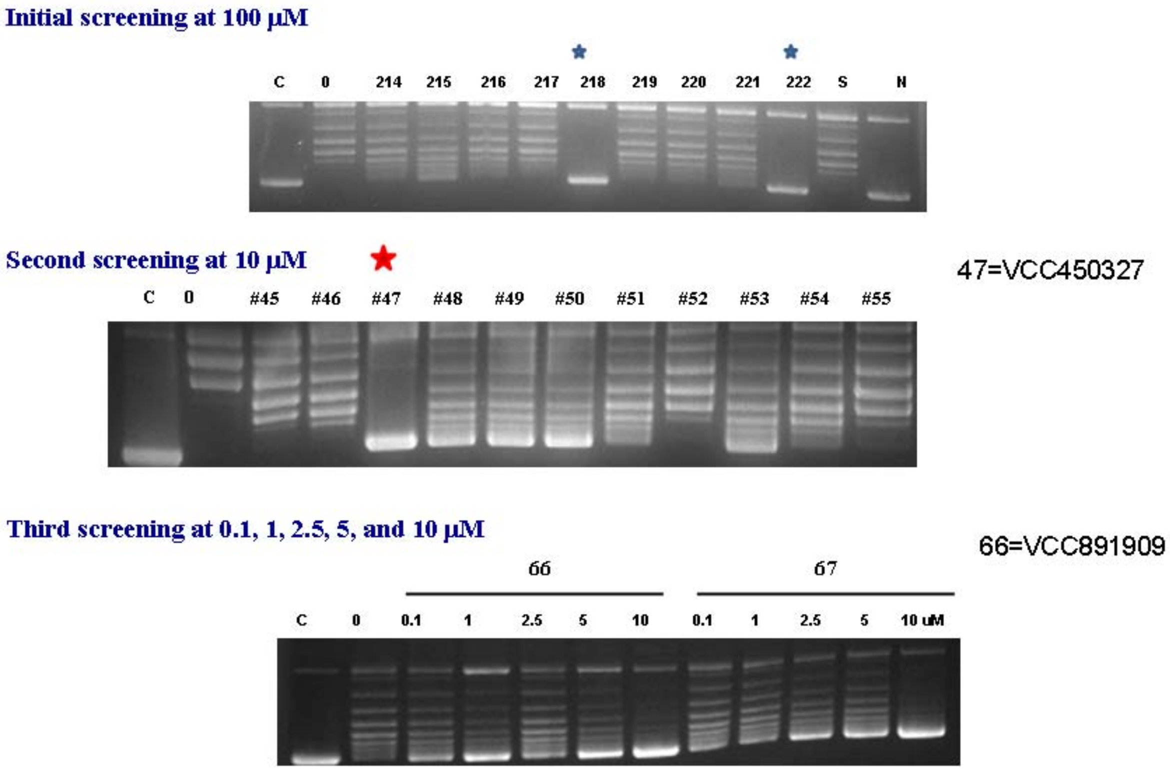

Supplement: S3 Fig — (PDF) [file pone.0202749.s003.pdf]
