# Supplementary material for: Characterization of new, efficient Mycobacterium tuberculosis topoisomerase-I inhibitors and their interaction with human ABC multidrug transporters
Source: PLoS One. 2018 Sep 5;13(9):e0202749. doi: 10.1371/journal.pone.0202749 (PMC6124754; doi:10.1371/journal.pone.0202749)

S4 Fig. Toxicity of the investigated compounds in H37Rv Mtb strains

The toxicity of the compounds in the virulent H37Rv Mtb strain was determined by growth inhibition tests as described in the Methods. Growth inhibitory potentials of the compounds were primarily screened at 20 μM, and for the effective compounds the minimum concentration providing 90% growth inhibition (MIC90 value) was determined from dose-response viability curves (20 to 0.04 μM). The curves presented here were the basis for MIC90 determinations shown in Table 2.

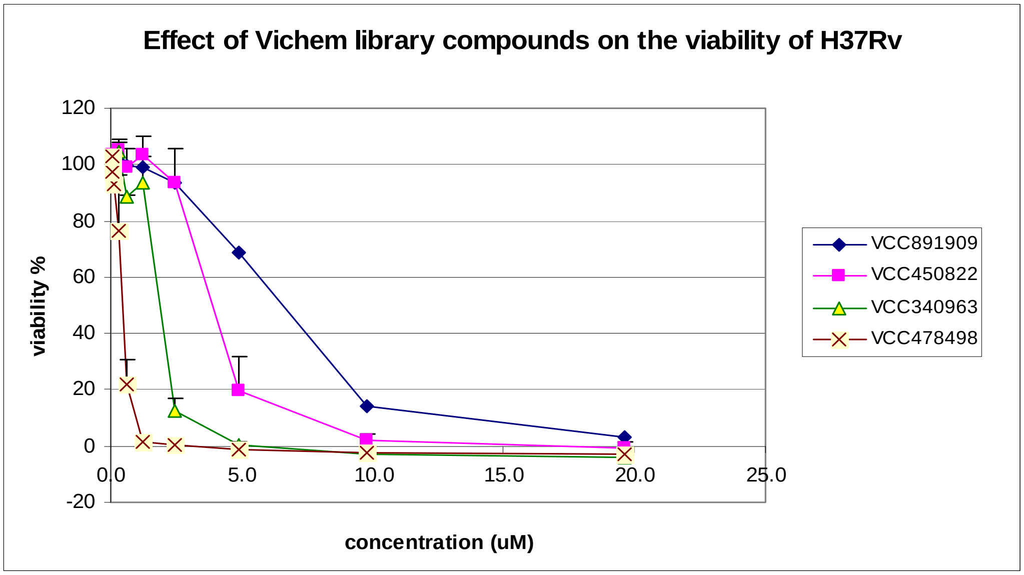

Supplement: S4 Fig — (PDF) [file pone.0202749.s004.pdf]
