# Supplementary material for: Characterization of new, efficient Mycobacterium tuberculosis topoisomerase-I inhibitors and their interaction with human ABC multidrug transporters
Source: PLoS One. 2018 Sep 5;13(9):e0202749. doi: 10.1371/journal.pone.0202749 (PMC6124754; doi:10.1371/journal.pone.0202749)

S5 Fig. Effects of the Vichem MtTopo-I inhibitors on the ATPase activity of the human ABCG2 and ABCB1 transporters.

Purified Sf9 membrane vesicles contained high levels of ABCG2 or ABCB1, as presented by the Western blots, in which ABCG2 was stained with BXP-21 (anti-ABCG2), and ABCB1 with JSB-1 (anti-ABCB1) monoclonal antibodies, respectively, and the bands detected by enhanced chemiluminescence. On western panel m1-3 represents various membrane preparations. Sf9 membrane vesicles were used in the ATPase assays as described in the Methods section. Vanadate sensitive ATPase activities were measured in dose-response measurements. V_{max} values were determined from curves presented in the main text in Fig. 2. Basal ATPase activity was determined without any given drug. * labels significant change ($p<0.05$, Student's test) between basal and V_{max} values.

VCC identity numbers	**ABCB1**		**ABCG2**	
	V_{max}	at conc.	V_{max}	at conc.
**verapamil**	16.7±0.4*	12.5 μM		
**quercetin**			38.5±1*	2 μM
**VCC891909**	7.8±1.2	3 μM	25.3±5	0.01 μM
**VCC979812**	15.8±2*	12.5 μM	22±3	0.4 μM
**VCC389777**	14.4±1.5*	12.5 μM	32.0±3.6*	0.4 μM
**VCC450327**	9.6±1.3	0.19 μM	27.2±0.8	0.4 μM
**VCC450822**	10.5±1.5*	0.19 μM	23.3±2	2 μM
**VCC340963**	7.9±1.2	0.19 μM	22.2±4	10 μM
**VCC478498**	7.2±1.8	6.25 μM	21.5±2.4	2 μM
**basal**	5.7±0.7		22±1.4	

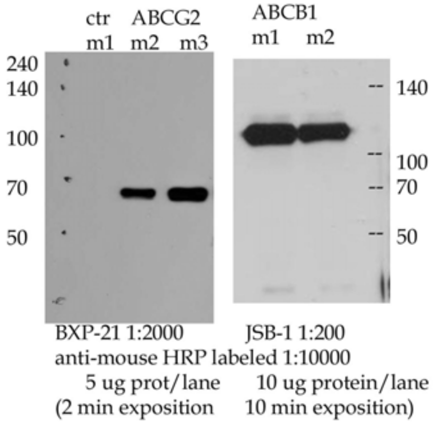

Supplement: S5 Fig — (PDF) [file pone.0202749.s005.pdf]
