# Supplementary material for: Characterization of new, efficient Mycobacterium tuberculosis topoisomerase-I inhibitors and their interaction with human ABC multidrug transporters
Source: PLoS One. 2018 Sep 5;13(9):e0202749. doi: 10.1371/journal.pone.0202749 (PMC6124754; doi:10.1371/journal.pone.0202749)
Supplement: S6 Fig — (PDF) [file pone.0202749.s006.pdf]

S6 Fig. Summary table for the results of the assays

0: no observed effect, +: effect observed in the assay. In the ATPase assay showing only inhibition, the - sign is applied. N.A.: not applicable. See the main manuscript text for further details.

VCC identity numbers	Toxicity in H37Rv assay	Toxicity in mammalian cell lines	ABC transporter ATPase assay		ABC transporter transport assay		ABC transporter modified cytotoxicity	
			ABCB1	ABCG2	ABCB1	ABCG2	ABCB1	ABCG2
**VCC891909**	+	0	0	0	0	0	N.A.	N.A.
**VCC979812**	0	0/slight tox.	+	0	+	+	0	0
**VCC389777**	0	0	+	+	+	+	N.A.	N.A.
**VCC450327**	0	0	0	0	+	+	N.A.	N.A.
**VCC450822**	+	+	0	0	0	+/-	0	0
**VCC340963**	+	+	0	0	+	+	0	0
**VCC478498**	+	+	0	-	+	+	0	0
